# Early Childhood Caries and Sleep Disorders

**DOI:** 10.3390/jcm12041378

**Published:** 2023-02-09

**Authors:** Ana Arroyo Buenestado, David Ribas-Pérez

**Affiliations:** 1 Faculty of Dentistry, University of Seville, 41004 Sevilla, Spain; 2 Department of Pediatric Dentistry, University of Seville, 41004 Sevilla, Spain

**Keywords:** early childhood caries, sleep disorders, obstructive sleep-disorders breathing

## Abstract

Obstructive sleep-disordered breathing (oSDB) comprises a set of breathing disturbances when the individual is asleep due to partial or complete upper airway obstruction. Modifying or risk factors are the anatomy, the size and shape of the airway, muscle tone, central nervous system responses to hypoxia, etc. In children, this is associated with poor school performance and reduced memory and learning abilities. In addition, increased levels of blood and lung pressure and cardiac alterations have been reported in children with sleep disturbances. On the other hand, Early Childhood Caries (ECC) is defined as the presence of one or more decayed primary teeth (cavities) of children under the age of 5. This study aimed to establish the possible relationship between sleep disorders and ECC by means of validated surveys and determined whether the results obtained coincide with the available literature. Our results found that up to 24.5% of children with a high risk of caries present regular nasal congestion, while this finding is only present among 6% of children with a low risk of caries (*p* = 0.041). The dmft index remains significantly linked to this occasional congestion, but the association depends on the patient’s level of risk (*p* = 0.008); increasing with a high increasing risk of caries. As a conclusion, the risk of early childhood caries could correlate to a specific sleep change such as occasional snoring.

## 1. Introduction

Obstructive sleep-disordered breathing (oSDB) is a collective term for breathing alterations caused by a partially or fully obstructed upper airway while a person is asleep [1]. The severity of the condition can range from snoring to obstructive apnea; (when the apnoea-hypopnoea index is >1.0/h) [1,2,3].

It can occur in adults and children, although both conditions course distinctly due to their different characteristics. The incidence among children is up to 25% in its mildest forms (snoring) and 1–5% in its most severe forms, also known as Obstructive Sleep Apnea Syndrome (OSAS) [1]. Establishing its precise prevalence is difficult, given that it varies by geographical region, as do the associated complications and the different diagnostic criteria used [4,5,6].

It has been demonstrated that OSAS causes intermittent episodes of hypoxemia and hypercapnia, interrupted sleep and frequent awakenings [3,4,6], accompanied with snoring, mouth breathing, apnea, daytime drowsiness, and large-sized tonsils [3,5].

Probably caused by interrupted sleep, people experience changes and alterations to behaviour and neurodevelopment [5,6], which increases stress levels at a biochemical and cell level, particularly in the frontal cortex [3]. Among children, this is associated with poor performance at school and lower memory and learning abilities. Furthermore, raised blood and lung pressure, along with heart changes (during systole and diastole), have been reported among children with sleep disorders, as well as atherosclerosis-induced changes during adulthood [7]. These effects appear to be reversible, and the sympathetic nervous system regains balance if the pathology is treated correctly.

During childhood, oSDB can be defined as a chronic multifactorial condition associated with genetic, anatomical, and pathophysiological changes [3]. It is caused by a change in homeostasis of the factors responsible for ensuring airway permeability and those causing its collapse. Additionally, the modifying or risk factors are the anatomy, size, and shape of the airway, muscle tone, central nervous system responses to hypoxia, hypercapnia, and the effects on sleep and awakening episodes [6]. As opposed to adults, childhood OSAS is mostly caused by tonsillar hypertrophy. The highest incidence among this age group is recorded between 2 and 8 years old, coinciding with peak tonsil growth [3].

Other potential factors linked to altered sleeping patterns among both adults and children include a person’s cranial-facial characteristics, allergic rhinitis [8,9], asthma, muscle tone, exposure to tobacco smoke, and low socio-economic statuses [5,6], although contrasted evidence is required to confirm these causal relationships [10,11,12,13].

oSDB diagnosis occurs using information obtained from the medical history and anamnesis, the clinical examination, and specific diagnostic tests [6]. Suspicion must be confirmed via an objective and validated test [14], such as polysomnography (PSG), which is considered the “gold standard” test because it provides objective information about sleep characteristics and breathing and heart patterns during sleep [4].

Caries is currently defined as a chronic imbalance in the demineralisation of the tooth’s surface caused by the accumulation of sugar-dependent cariogenic oral flora or biofilm [15]. The main risk factors for caries to appear are oral flora and diet, the mechanical removal of plaque and fluoride exposure, and a person’s own susceptibility (socio-economic-cultural and behavioural factors or local defects) [16,17,18]. 

Early Childhood Caries (ECC) is defined as the presence of one or more primary teeth with decay (with or without cavities), missing teeth (due to decay), or filled teeth among children aged under 6 years old. The definition of Severe Early Childhood Caries (SECC) is applied to children aged under 3 years old with any sign of decay and 4 or more decayed teeth, missing teeth, or filled teeth for children aged 3 years old, 5 for children aged 4 years old, or 6 for children aged 5 years old (American Academy of Pediatric Dentistry (AAPD), 2020) [18]. The AAPD created a guideline for identifying a child patient at risk of caries, which was also used for this study to evaluate its predictability and to determine whether its results are corroborated [18]. 

There have been several studies that attempted to demonstrate the relationship between the conditions described above. There is no clear association between them, although it is true that there are shared etiological factors in both disorders [19,20] that may act as confusion factors. Based on this, the objectives of this study were to establish a possible link between sleep disorders and ECC using validated surveys and to determine whether or not the results agree with the available literature. There are two parts to the study, the clinical study and recording of the different parameters for evaluation and a statistical analysis, to establish the link between them. 

## 2. Materials and Methods

### 2.1. Study Type, Setting, and Inclusion and Exclusion Criteria

The clinical examination of this analytical cross-sectional study took place at a private dental clinic (Clínica María Isabel Rodríguez) and was performed on 80 healthy children.

Inclusion and exclusion criteria were children aged 2–5 years old, who did not regularly consume medication and did not have any orthopaedic treatment. They had to be children without medical conditions requiring medication for respiratory problems. Furthermore, their body mass index (BMI) had to be less than 95% for their age and gender. 

Their clinical condition was reviewed, and the children suspected to have interproximal decay and whose behaviour permitted it received X-rays using bitewings with a mirror, probe, air, and suction cannula. 

The private dental clinic is located in Pozoblanco, Córdoba, whose estimated population was 17,156 inhabitants, according to the Instituto de Estadística y Cartografía de Andalucía, including 3328 people under 20 years old. There was no specific information about the population under this age; thus, we decided to ask public and private education centres in the village, and we estimated that the number of children aged 2 to 5 years old was between 400 and 450. For the calculation of the sample size, taking into account this population, we used a confidence level of 80% with an error rate of 6.5%.

With this information, using a written call, we asked 250 randomly selected children to participate in our study. Simple random sampling was carried out, with patients from a given list using specific software for this purpose. Finally, just 120 of those children answered positively and joined our study. After the clinical exploration, 40 children were rejected because they did not meet the inclusion criteria.

### 2.2. Dental Assessment

All patients had the same clinical examiner (AAB), with the same methodology used on them all to avoid bias. In order to avoid possible errors arising from multiple observers, it was decided to carry out the scanning for data collection through the work of a single examiner. However, it was of utmost importance to ensure uniformity within the observations made by this single examiner and consistency of the observations made by this single examiner. Therefore, in order to measure the consistency of her observations, the examiner was subjected to a so-called intra-observer calibration, obtaining the percentage of agreement with the Kappa test (0.85).

Decay was diagnosed and recorded based on pre-established WHO ECC criteria [21]. However, given that this record does not contemplate teeth missing due to caries (mt) and that we recorded several children with teeth lost early due to caries in our sample, we changed the index to dmft.

### 2.3. Study Period and Questionnaire

The study was carried out during the first three months in 2021. The mother, father, or main caregiver was asked to fill out a validated questionnaire (Spanish version of Paediatric Sleep Questionnaire—PSQ) (Appendix A) about their child’s sleep habits and characteristics. This questionnaire aimed to show the possible changes linked to three main aspects: snoring, excessive daytime sleepiness, and behavioural changes. 

Lastly, a questionnaire based on the AAPD guideline was filled out by the clinical examiner with the present mother, father, or main caregiver, about the caries risk factors for each child in order to individually determine their caries risk independently of the caries lesions that were recorded. 

### 2.4. Statistical Analysis

The SPSS 15.0 programme (SPSS Inc., St Halley Statistics, Valencia, Spain) was used to interpret the data. Based on the defined objectives, the dmft index was accepted as the primary investigation variable, given that the aim was to study its relationship with sleep disorders and with caries risk evaluation. A Kolmogorov-Smirnov test was applied to contrast the fit of the dmft index to the normal distribution, with a negative result (*p* < 0.05). The good sample size would generally enable the use of a comparison of means tests. However, the prevalence of most of the PSQ disorders is very low; thus, applying non-parametric tests to contrast distributions rather than means was recommendable. Bivariate analysis covers every statistical contrast required to meet the investigation’s objectives.

- The Mann–Whitney U test for two independent samples (MW); the Kruskal-Wallis extension for more than 2 samples was also used.
- Spearman’s non-linear correlation coefficient.

A two-way ANOVA linear model was estimated to study the differences of the mean dmft index depending on the presence/absence of a certain sleep disorder and caries risk level. The aim of this adjusted model was to conclude whether or not a sleep disorder is actually affecting dmft or is a result determined by a person’s level of caries risk. Although this is a parametric form of analysis, it is the only possible option, as stratification meant reducing the samples to very small groups (n ≤ 16), with the subsequent loss of statistical power. A 5% significance level was used in the analysis (α = 0.05). 

## 3. Results

The sample for the investigation comprised 80 children aged 2 to 5 years old, mainly aged 4–5 years old (77.6%). There were 40 boys (50%) and 40 girls (50%) (Table 1).

### 3.1. Sleep Disorders

When analysing the sleep disorder results, they can be divided into the same 3 aspects as the test. As such, when it comes to a child’s behaviour during the night and sleep, almost half of the sample occasionally snore (43.8%) and/or speak (46.3%). Furthermore, one of every three children presents restless sleep and extremity movement while asleep, as well as finding it hard to fall asleep. The same proportion of the sample wakes up to go to the bathroom at least once. Additionally, 1/3 of all the studied children sleep with their mouths open and have mouth dryness upon waking. 

When it comes to sleep behaviour during the day, over 25% have a nap during the day, with morning tiredness and difficulties getting up also common (27% and 32%, respectively). Almost 100% of the sample (97.5%) still have their tonsils, and only 9% have been medically diagnosed as overweight. 

Lastly, when the child’s behavioural problems were studied, most children did not pay attention to details or tasks, they lost things, or they were easily distracted (Table 2).

### 3.2. Early Childhood Caries

The second aspect studied was the dmft index, a variable response key to the child’s oral health status. The mean dmft index is 1.73 ± 2.34, ranging from 0 to 8. However, the median is 0, i.e., over half of the children have a dmft = 0. 

**Figure 1:**
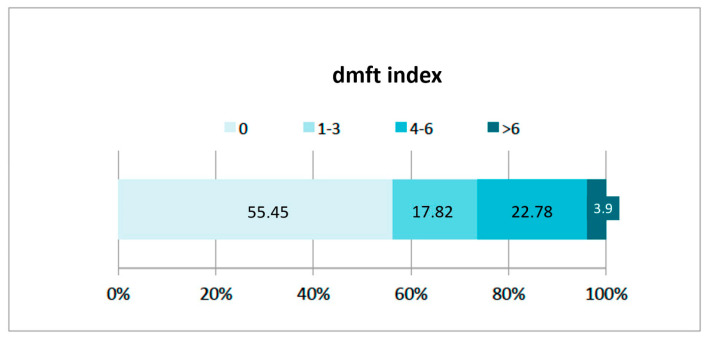
Percentage of children and distribution per dmft.

When this index was investigated further, 55.4% had a zero dmft, 17.82% had between 1 and 3, 22.78% between 4 and 6, and only 3.95% had an index over 6 (Table 3) (Figure 1).

### 3.3. Caries Risk Evaluation

Among the caries risk factors, most parents or main caretakers had caries (55%), and up to half of the sample eats sugary foods, drinks, or snacks on a daily basis (52.5%). Given that most children will continue to see a dentist, they are considered to have a dental household when they are given health and diet control guidelines, therefore brushing with toothpastes containing fluoride (66.3%). Up to when this study’s examination was performed, however, many children had not yet seen a dentist, meaning untreated cavity lesions are also very common (42.5%). However, brushing is unassisted on most occasions, and plaque is observed in over 80% of children. 

To sum up, 61% of children are at high risk of caries (Table 4).

### 3.4. ECC and Sleep Disorders

On linking both values, we found that the mean dmft index was 2.63 ± 2.50 for occasional snorers and 1.02 ± 1.96 for non-snorers. The distributions were significantly different in both groups (*p* = 0.001). There were also statistically significant relationships between the dmft index and noisy or deep breathing (*p* = 0.029) and sleepwalking and nightmares (*p* = 0.009 and *p* = 0.033, respectively). 

It stood out that the ECC indices were higher among children who go to bed and get up late, children that wake up tired, or those that find it hard to wake up. This relationship is also statistically significant (Table 5).

### 3.5. The Risk of Caries and ECC

When attempting to link the risk factors for early childhood caries and lesion appearance, we found that too much sugar exposure, the detection of incipient caries lesions or non-cavitated lesions, and cavitated lesions are very closely related and statistical factors (*p* < 0.001). The dmft index is strong and significantly correlates with the overall determination of the level of caries risk estimated for the child (*p* < 0.001).

### 3.6. Risk of Caries and Sleep Disorders

In order to determine whether or not caries risk factors are themselves confounders in the main relationship under study (oSDB-ECC), whether or not more sleep problems actually occur among children with moderate or high level of caries risk was checked, given that they influence both variables. Therefore, we found that up to 24.5% of children with a high risk of caries present regular nasal congestion, while this finding is only present among 6% of children with a low risk of caries (*p* = 0.041). Additionally, the eight children that could possibly sleepwalk, as indicated by their parents, are in the high-risk group for caries (*p* = 0.022). Lastly, the later a child goes to bed and the harder they find it to get up in the morning, the more often caries is observed (*p* = 0.019 and *p* = 0.002, respectively) (Table 6).

### 3.7. Final Results

**Figure 2:**
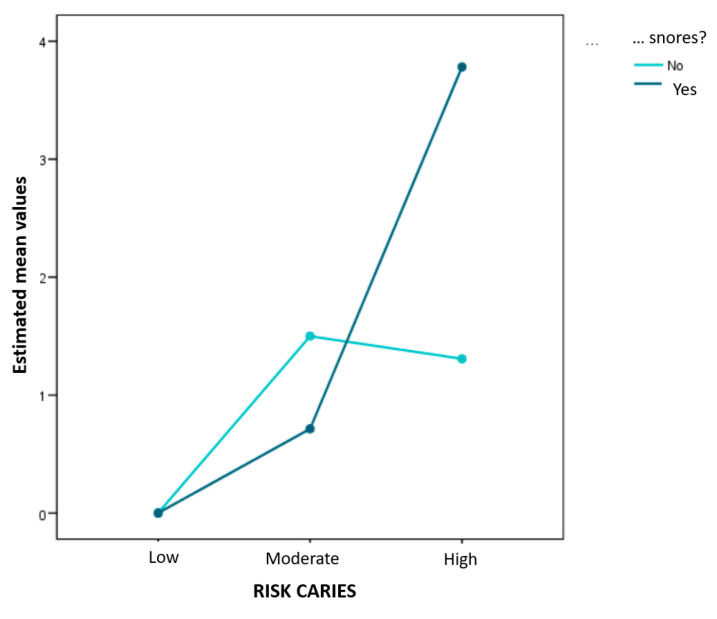
Mean values of the index for the combinations of both factors. When the level of risk is low or moderate, the dmft index is similar between occasional snorers and other children. However, if the level of risk is high, occasional snorers raise the index strongly.

Finally, the ANOVA model was applied to clarify the extent to which sleep disorders affect ECC or whether or not it is simply that those common risk factors are confounding. The results showed that the dmft index remains significantly linked to occasional snoring, but the association depends on the patient’s level of risk (*p* = 0.008), increasing with an increasing risk of caries (Table 7) (Figure 2).

However, when it comes to the other analysed variables that initially showed a potential link, it is now clear that it does not exist, and that the risk of caries itself explains the dmft index observed in the patient in question (Table 7).

## 4. Discussion

Before starting to discuss the literature, it is important to note that there is not any current evidence regarding a possible link between the factors under analysis. When studied in order to understand the pathology’s characteristics, we found that the most commonly found symptoms and signs among patients with oSDB are snoring, mouth breathing, apnoea, daytime drowsiness, and large tonsil size [4,5]. 

On behalf on caries data, when the results of our work were compared with those obtained in the only population-based study carried out in Spain in 2007 for pre-school children [22], they differed slightly. Thus, in the present study, higher dmft indexes were recorded (1.73 ± 2.34 in children aged 2–5 years vs. 0.52 and 0.76 in children aged 3 and 4 years, respectively), although these indexes are not completely comparable.

In 2015, Nouf et al. [23] analysed through a case-control study the oral characteristics of 30 children with well-diagnosed OSAS and/or snorers and compared them with 30 healthy children (only related by sex and age), aged 3–8 years. 

Contrary to what was expected, their results showed that the subjects in the study group showed lower caries rates and better oral hygiene (less gingivitis) than the control group. Their results, however, cannot be fully compared with those obtained in our work because of the type of study, the enormous difference between an OSAS diagnosis and nocturnal snoring, and the poor inclusion and exclusion criteria to which they subjected the patients, which could be closely related to the risk of caries. 

Similarly, but more recently, Davidovich and coworkers [24] found a negative relationship between OSAS and caries in 2022. Although this was another case-control study with a small sample size, older children, and a medical diagnosis of OSAS, the dental and gingival health of the patients in the study group was not related to sleep disturbance or severity of sleep disturbance. 

It is characteristic that the authors found a higher microbiological count (responsible for caries) in the study patients when compared to the control ones. The multifactorial nature of caries and its intimate relationship with sugar consumption (which is not analysed here) would explain why both parameters were not related in this study. 

The articles found a link between the presence of oSDB and a higher predisposition for dental changes due to a high prevalence of mouth breathing. Secondary to this, these patients may experience higher rates of xerostomia, and given that OSAS is linked to higher rates of obesity and low socio-economic statuses, we can also consider that the rate of caries and periodontal disease is higher among patients with sleep disorders [19,20,25]. Lastly, the treatment used to resolve oSDB for mild to moderate cases, whether using an orthopaedic-orthodontic apparatus, CPAP, or a drug treatment, could also represent an additional risk factor for oral disease due to the respective increased plaque retention and oral dryness that occurs [19,20].

Grillo and La Mantia [19] analysed the oral health of children and young people (aged 8–17 years old), but it only included healthy patients that did not wear any orthodontics. Furthermore, they also evaluated sleep alterations using the validated PSQ questionnaire. They analysed several aspects of oral health, not just caries. The authors reached the conclusion that higher rates of caries occur among patients with sleep disorders, particularly patients with oSDB. However, the cause for this link was not fully described, and they stated that it may derive from both pathologies sharing risk factors, such as mouth dryness.

Additionally, Acar and Türkcan [26], in their case-control study, tried to relate OSAS and its severity to oral health status. Specifically, similar to our study, they tried to relate this sleep disorder and dmft. They based their study on the salivary decrease observed in patients with OSAS, especially those with CPAP, which causes a greater accumulation of plaque and, consequently, a higher risk of caries. The study concluded that the intensity with which OSAS occurs has no significant relationship with oral health status, but the duration of OSAS does. The results obtained cannot be fully compared with our work, since the population studied was completely adult (mean age 50 years), the type of study also differs, and not all caries risk factors were taken into account; only the socioeconomic and educational level of the patient was analysed. In addition, other possible causes of salivary decline such as medication intake or systemic diseases were also not analysed. 

Additionally, Tamasas et al. [20] stated that sleep disorders and oSDB can cause oral changes, which influence the development of mouth breathing and xerostomia, and that these predispose the appearance of caries and other oral changes, such as gingivitis or periodontitis among children aged between 8 and 17 years old with a clear oSDB diagnosis. Among other parameters, they concluded that the number of caries lesions in permanent teeth among patients with oSDB was significantly higher. However, this difference was not observed in primary teeth. It is likely that this conclusion was based on the fact that the studied sample was aged 12–14 years old on average, i.e., with the teeth almost fully replaced. Therefore, their results cannot be fully compared to those in our study. 

A more recent cross-sectional study, by Blumer and coworkers [27], attempted to relate, in contrast to our study, possible oral characteristics that could help in the early identification of sleep disorders. For this purpose, it analysed the oral characteristics of a population group with a mean age higher than that included in the present study (4–12 years) and related them to the findings obtained by the same questionnaire (PSQ). Among these possible findings, however, was not an increase in the risk of caries; therefore, they did not conclude that this could not be interpreted as a risk indicator or a consequence of the previous one. 

To sum up, although these studies cannot be fully compared with the results we achieved, it could be stated that they more or less match our statements, whereby oral pathologies in general, and caries in particular, could show a higher incidence due to shared risk factors with sleep changes, but to a lesser extent because they themselves are a risk factor among children.

The biases of this study include the lack of analysis of the socio-economic statuses and education levels of the family of the studied children or the level of mouth dryness stated by patients or the family as caries risk factors. The xerostomia, associated with mouth breathing and snoring at night, may also be conditioned by a range of pathological or drug factors, although we cannot always identify this using the medical history, and would therefore need a complementary study [28,29,30,31,32,33,34].

## 5. Conclusions

The analytical observational descriptive study showed that the risk of early childhood caries could correlate to a specific sleep change such as occasional snoring. On the other hand, it was observed that the presence of caries is directly related to the presence of risk factors, such as hygiene and dietary habits, and a prior experience of caries. Therefore, although some sleep disorder aspects are more common among children with high dmft indexes, they originate because they share risk factors. The level of risk of caries is in itself enough to explain the patient’s oral health status for most of the studied cases, overshadowing sleep problem effects. The other consulted articles cannot be contrasted with the conclusions due to differences in the study structure.

## Figures and Tables

**Table 1:** Demographic distribution (age and gender).

		Population	Percentage
Gender	Total	80	100%
	Boys	40	50%
	Girls	40	50%
Age	2	9	11.3%
	3	8	10.0%
	4	25	31.3%
	5	37	46.3%

**Table 2:** Sample distribution of sleep disorders and effects on the behaviour.

		Population	Percentage
Behaviour at night and sleep	Snoring	35	43.8%
	Speaking	37	46.3%
	Deep breathing	22	27.5%
	Sleeping with mouth open	28	43.8%
	Stop breathing	4	5.0%
Behaviour during the day	Nap	22	27.5%
	Morning tiredness	19	23.8%
	Difficulties getting up	26	32.5%
	Tonsils operation	2	2.5%
	Overweight	7	8.8%

**Table 3:** Dmft per age (mean and SD.) * Excluded a 10-month-old child.

	n	dmft x	SD
Total	80	1.73	2.34
<3 years *	17	0.85	1.96
4 years	25	1.68	2.21
5 years	37	2.22	2.54

**Table 4:** Distribution by caries risk evaluation.

	n	%
Total	80	100%
Low caries risk	16	20%
Moderate caries risk	15	19%
High caries risk	49	61%

**Table 5:** Relationship between sleep disorders and the dmft. Mean value of dmft and the sleep disorder with its standard deviation (SD).

Nº Question		n (Yes/No)	dmft x (Yes) SD	dmft x (No) SD	*p* Value	r Value
A1	snores at any time	80 (35/45)	2.63 ± 2.50	1.02 ± 1.96	0.001 *	
A4	snores loudly	80(63/17)	2.56 ± 2.58	1.41 ± 2.11	0.072	
A5	heavy breathing	80(58/22)	2.50 ± 2.61	1.43 ± 2.18	0.029 *	
A24	mouth open during day	80(64/16)	0.92 ± 1.50	1.80 ± 2.38	0.247	
A25	dry mouth on awakening	80(49/31)	1.38 ± 2.14	2.06 ± 2.49	0.335	
A32	nocturnal eneuresis	80(67/13)	2.09 ± 2.70	1.72 ± 2.31	0.741	
A33	sleepwalking	80(72/8)	3.63 ± 2.50	1.51 ± 2.24	0.009 *	
A35	nightmares	80(61/18)	2.83 ± 2.74	1.39 ± 2.14	0.033 *	
B1	unrefreshed in morning	80(61/19)	2.89 ± 2.94	1.36 ± 2.01	0.038 *	
B6	hard to wake up	80(54/26)	2.77 ± 2.67	1.22 ± 2.00	0.009 *	
B22	obesity	80(66/14)	2.36 ± 2.92	1.59 ± 2.20	0.279	
C3 **	does not listen	80(43/19/18/0)		1.49 ± 2.14	0.293	0.12
C5 **	difficulty organizing	80(53/15/12/0)		1.58 ± 2.26	0.501	0.08
C8 **	easily distracted	80(40/29/11/0)		1.58 ± 2.31	0.309	0.12
C10 **	fidgets	80(59/11/10/0)		1.31 ± 2.02	0.344	0.13
C14 **	on the go	80(45/22/11/2)		1.58 ± 2.17	0.476	0.08
C18 **	interrupts	80(43/25/12/0)		1.33 ± 2.12	0.093	0.19

* The Mann–Whitney test evaluates whether the distribution between the two groups is significant or not, with statistical significance for values of *p* < 0.05. ** The questions in group C establish more than two answers (Yes/no) by establishing the frequency of situations in four groups (never/sometimes/many times/always). In this group, we studied not only the *p* value but also the Spearman’s r correlation coefficient, which in all cases had a correlation of less than 0.25, indicating a weak correlation.

**Table 6:** Sleep disorders according to caries risk level; Mann–Whitney (MW) test results and Spearman’s r correlation coefficient.

Nº Question		*p* Value	r Value
A4	snores loudly	0.861	
A5	heavy breathing	0.495	
A6	trouble breathing	0.366	
A22	nose congestion	0.041 *	
A24	mouth open during day	0.500	
A25	dry mouth on awakening	0.340	
A32	nocturnal eneuresis	0.176	
A33	sleepwalking	0.022 *	
A49	bedtime	0.019 *	0.26
B1	unrefreshed in morning	0.078	
B6	hard to wake up	0.002 *	
B9	delayed growth	0.743	
B22	obesity	0.570	
C3	does not listen	0.716	0.04
C5	difficulty organizing	0.050	0.22
C8	easily distracted	0.006 *	0.31 *
C10	fidgets	0.066	0.21
C14	on the go	0.231	0.14
C18	interrupts	0.108	0.18

* statistical significance for values of *p* < 0.05.

**Table 7:** Results of the 2-factor ANOVA model: Relationship dmft with each question and caries risk level (Table 7). For question A1, dmft shows a significant relationship with occasional snoring, and this association is also dependent on the patient’s caries risk level (*p* = 0.008).

Relation to dmft (ANOVA Model)			*p*-Value
A1	(ocasional snoring)	Risk level of caries	0.008 *
A5	(noisy breathing)	Risk level of caries	0.659
A33	(sleepwalking)	Risk level of caries	--
A35	(nightmares)	Risk level of caries	0.780
A49	(bedtime)	Risk level of caries	0.734
B1	(tiredness on awakening)	Risk level of caries	0.159
B6	(difficulty in waking up in the morning)	Risk level of caries	0.142

* statistical significance for values of *p* < 0.05.

## Data Availability

Data can be provided by the authors if it´s required.

## Appendix A. Reduced Version of Pediatric Sleep Questionary

A. Behaviour at night and sleeping.				
While sleeping, does your child...				
A2…snore more than half the time?		S	N	NS
A3…always snore		S	N	NS
A4…snore loudly?		S	N	NS
A5…have “heavy” or loud breathing?		S	N	NS
A6…have trouble breathing, or struggle to breathe?		S	N	NS
Have you ever…				
A7…seen your child stop breathing during the night?		S	N	NS
Does your child				
A24…tend to breathe through the mouth during the day?		S	N	NS
A25…have a dry mouth on waking up in the morning?		S	N	NS
A32…occasionally wet the bed?		S	N	NS
B. Behaviour during the day and other problems.				
Does your child…				
B1…wake up feeling unrefreshed in the morning?		S	N	NS
B2…have a problem with sleepiness during the day?		S	N	NS
B4…Has a teacher or other supervisor commented that your child appears sleepy during the day?		S	N	NS
B6…Is it hard to wake your child up in the morning?		S	N	NS
B7…Does your child wake up with headaches in the morning?		S	N	NS
B9…Did your child stop growing at a normal rate at any time since birth?		S	N	NS
B22…Is your child overweight?		S	N	NS
C. Please, mark with an X	Never	Sometimes	Many times	Always
This child often…				
C3…does not seem to listen when spoken to directly.				
C5…has difficulty organizing task and activities				
C8…is easily distracted by extraneous stimuli				
C10…fidgets with hands or feet or squirms in seat				
C14…is ‘on the go’ or often acts as if ‘driven by a motor.				
C18…interrupts or intrudes on others (e.g., butts into conversations or games)				
